# Effects of illicit drugs on structural and functional impairment of testis, endocrinal disorders, and molecular alterations of the semen

**DOI:** 10.22038/ijbms.2021.53326.12002

**Published:** 2021-07

**Authors:** Zohreh Nazmara, Babak Ebrahimi, Pouran Makhdoumi, Leila Noori, Seyed Amirhosein Mahdavi, Gholamreza Hassanzadeh

**Affiliations:** 1 Department of Neuroscience and Addiction Studies, School of Advanced Technologies in Medicine, Tehran University of Medical Sciences, Tehran, Iran; 2 Department of Anatomy, School of Medicine, Tehran University of Medical Sciences, Tehran, Iran; 3 Students Research Committee, Kermanshah University of Medical Sciences, Kermanshah, Iran; 4 Department of Biomedicine, Neurosciences and Advanced Diagnostic, University of Palermo, Palermo, Italy; 5 Department of Anatomical Sciences, School of Medicine, Babol University of Medical Sciences, Babol, Iran; 6 Legal Medicine Organization, Tehran, Iran

**Keywords:** Amphetamines, Cocaine, Illicit drugs, Male infertility, Male reproductive system, Marijuana, Opioids

## Abstract

Illicit drug use is growing among young people, which is one of the major problems in today’s society that can be associated with many medical issues, including infertility. Amphetamines, cocaine, opioids, and marijuana are the most common and the most used illicit drugs worldwide. The purpose of this review was to collect as much literature as possible about the impact of illicit drugs on male fertility and summarize their valuable data. Original studies and reviews were collected by searching the keywords “illicit drugs (all kinds of that) and male infertility”. The obtained information was also categorized based on the content of the “Infertility in the Male” book. Almost all studies suggested that taking all kinds of illicit drugs with the effects on different parts of the male reproductive system can result in subfertility or complete infertility in the consumers. Although the data in this field are not decisive and there are some confounding factors in human studies, it can be inferred that the use of any illicit drug with an effect on male sexual health reduces fertility potency. Therefore, it is recommended that couples, who are planning to conceive, avoid taking any illicit drugs before and during treatment.

## Introduction

Infertility is defined as failure to achieve a pregnancy after a year of unprotected intercourse. Considering the growing number of infertile couples, and psychological, social, and economic problems stemming from it, this social-medical condition has intrigued researchers in recent years. According to the latest reports, more than 50 million couples worldwide suffer from infertility. World statistics suggest male infertility is 2.5–12 percent, which accounts for 20–70 percent of couples (1). Primary testicular disease (2), endocrine impairment (3), erection and ejaculation disorders (4), infection (3), and alteration of semen characteristics (5) were considered as main factors in the etiology of male infertility. Considering the effect of the niche of the spermatozoa on their survival and function, spinal cord injury (6), testicular trauma (7), stresses (8), and drugs (9) have a special place in men’s sexual capacity. The increasing growth of recreational drug use by young people in reproductive ages around the world has prompted researchers to investigate the adverse effects of illicit drugs on fertility. However, few *in vitro* and clinical studies have been conducted in this regard. 

For writing of this manuscript, we made a list of relevant keywords and phrases and then started searching studies in PubMed, Scopus, and Web of Science databases. The keywords that we used were: Illicit drugs, Male infertility, Male reproductive system, Amphetamines, Cocaine, Opioids, and Marijuana. In this review study, the effects of various illicit drugs on each of the infertility factors were elaborated separately.

**Primary testicular disease, the most common cause of male infertility**

In contrast to women, in men, gametogenesis defects are generally due to damage to the testicular tissue and are rarely associated with pituitary dysfunction. Primary testicular dysfunction results in reduction or complete stopping of spermatogenesis (10). Decreased testosterone production due to changes in Leydig cell function is one of the conditions causing the primary testicular disorder. Due to the reduced rate of spermatogenesis, men develop oligospermia or azoospermia. Although it is one of the most common causes of male infertility, about 65% of cases are idiopathic and asymptomatic infertile men (2).

It should be borne in mind that the disease is incurable and there is no known treatment to increase sperm cell numbers or change these conditions. Another important feature of the disease is the progressive process of decreasing quantity and quality of spermatozoa; azoospermia may occur in cases of severe oligospermia due to primary testicular dysfunction (3). Therefore, the prevention of primary testicular disease is the most important clinical approach. Drug abuse and exposure to its smoke can be potential contributors to the early testicular disease in male infants and male offspring of addicted parents (11). Although there are no laboratory and clinical studies examining addicted fathers and the sexual health of their offspring, the effect of morphine on the reproductive system of mice was age-dependent and its destructive effect has been reported more in immature mice and pre-adolescence (12). Studies on primary testicular disease are listed under three subtitles: deficiency in Leydig cell function, structural alterations of seminiferous tubules, and deficient testicle functions. The impact of illicit drugs on primary testicular disease is summarized in Table 1.

**Deficiency in Leydig cell function**

The alterations in Leydig cell activity is one of the issues discussed concerning the primary testicular disorder.

***Amphetamines***

 The Ras signaling pathway, which is the most important hormone transport pathway, is blocked by protein kinase A. Increased cyclic adenosine monophosphate (cAMP), which acts through this protein kinase in the testis of the amphetamine-treated rats, suggested the hypothesis of inhibition of testosterone transduction and release from Leydig cells in this group of rats (13).

***Opioids***

The results of Yilmaz *et al*. (1999) with the expression of healthy Leydig cells in the testis tissue of young (30–33 days old) Wistar rats treated with morphine showed that the decrease in testosterone was due to impairment of the Hypothalamus-Hypophyseal-Testicular (HPG) axis, and testosterone is reduced through the brain’s opioid system (14). This report indirectly ruled out the possibility of an association between primary testicular disease and morphine consumption. However, some researchers believe that the direct effect of opioids on testosterone levels is through opioid receptors in the testes (15). Pro-opiomelanocortin (POMCs) -derived peptides exist in the male reproductive tract of many animal species. Beta-endorphin has been observed as one of the major POMC-derived peptides in the adult testis extract. The increase of this peptide in mouse and hamster Leydig cells during adolescence and afterward indicates a relationship between beta-endorphin and steroid-producing capability (16). Due to the lack of opioid receptors at the Leydig cell surface and detection of opioid-binding regions in Sertoli cells, it has been hypothesized that the Leydig cell-derived opioids have a local effect on the regulation of steroids production. However, the importance of this mechanism and the function of opioid receptors in the production of testosterone is not fully understood. In humans, the decreased concentration of LH-independent testosterone in male heroin and methadone consumers could be due to the direct impact of the drugs on testicles (17). The adverse effects of opioids could be observed even after two years past withdrawal. Other observations reported on the study included oligospermia, asthenospermia, and sperm abnormal morphology plus an increase in the number of immature sperm cells (18). 

***Marijuana***

The presence of endocannabinoid system elements in the anterior pituitary and testicular tissues and the function of exogenous cannabinoids through this system alongside the destructive effect of marijuana on the HPG axis, spermatogenesis, and sperm functionality have been reported by some researchers (19). Decreased luteinizing hormone (LH) and testosterone secretion in cannabinoid receptor mutant mice and healthy mice via anandamide (a receptor agonist) has been shown. Cannabinoids with different origins can reduce testosterone secretion which results in spermatogenesis impairment. In humans, oligospermia was reported in over one-third of marijuana consumers (20).

**Structural alterations of seminiferous tubules**

Changes in the microarchitecture of seminiferous tubules lead to changes in the function of the tubules. 

***Amphetamines***

A short-term injection of methamphetamine to mice will induce apoptosis in seminiferous tubules (21).

***Cocaine***

A short-term or long-term cocaine abuse in immature and mature rats resulted in decreased diameter of seminiferous tubules, increased number of abnormal seminiferous tubules with degenerated cells by 50% to 60%, decreased cell adhesions, abnormal cellular structures, decreased quantity of germ cells, and reduced spermatogenesis (22). Other adverse effects include the presence of vacuoles and lipid droplets, giant mitochondria, and apoptosis in germ cells (23). 

***Marijuana***

Adverse effects of cannabis on dogs include basement membrane destruction, decreased diameter of seminiferous tubules, and germ cells’ quantity and quality reduction (24).

***Opioids***

Diameters of the seminiferous tubules and their epithelial height are considered two essential factors while studying the impact of toxins on spermatogenesis (25). Reduced epithelial thickness in seminiferous tubules was observed in rats treated using Iranian Kerack which is a layman name used for a new type of opioid (26). According to several studies, methadone (27), tramadol (28) and morphine (29) have the same impact on the seminiferous tubules in rats. In contrast, a study of morphine impacts on Wistar rats by Yilmaz *et al*. (1999) included a report on the normal structure of seminiferous tubules (14). The most important variables affecting the results of various studies include the type and purity of opioids, treatment dose, treatment duration, number of treatments per day and week, routes of opiate consumption, species, age, weight, and health conditions of the animal. 

Rats undergone the ecstasy treatment were reported with degenerated seminiferous tubules (30).

**Deficient testicle functions**

Major functions of the testicles include steroidogenesis (testosterone in particular) and spermatogenesis (31). The current section is conducted to study the adverse effects of various illicit drugs on testicular function.

***Cocaine***

Rats treated with cocaine were observed with deficient spermatogenesis, abnormal seminiferous tubes, cellular destruction, cell sloughing, and abnormal cellular structure (32). In human beings, cocaine abuse can induce cellular death of testicles (30).

***Marijuana***

A 2 mg/kg injection of delta-9-tetrahydrocannabinol (THC) to rats caused reduction in enzymatic functions of Sertoli and interstitial cells. The fact that testicular functions are deranged due to THC-induced decrease of gonadotropin level is supported by gonadotropin treatment which leads to improved enzyme functions of testicles (33). Injecting a high dose of THC is known to disturb several testicular enzymes such as β-glucuronidase, Alpha-glucosidase, acid phosphatase, and fructose-6- phosphatase (34). The outcome of marijuana treatment on rodents included increased abnormal morphology in sperm cells, mitosis and meiosis impairment during spermatogenesis, and increased rate of deformed sperm cells (20). However, the presence of cannabinoid receptors in sperm cells indicates the role of these agents in normal sperm function (35). In human studies, researchers (2015) reported the expression of CB1 (a cannabinoid receptor) in testicles, vas deferens, and sperm cells. Further in this study, it was determined that marijuana can affect spermatogenesis and mature sperm cells (36). 

***Opioids***

opioid-induced hypogonadism has been reported in animal experiments and clinical observations in a comprehensive review article (37). The presence of opioid receptors in testicles may be related to hypogonadism. Iranian heroin can impair spermatogenesis of mice, as well (15). Morphine and the derived drugs will cause a reduction in various cells of spermatogenesis cycles of rats (29). In humans, long-term, intrathecal injection of opioids would lead to hypogonadism, whereas androgen therapy could significantly improve the symptoms. A long-term, oral opioid consumption as to sedate non-cancer, chronic pain will cause hypogonadism in male consumers. Safarinejad *et al*. (2013) reported disturbing gametogenesis in male opioid addicts (38). Additionally, several authors have considered the presence of enkephalins in sperm cells and the probable role of these endogenous opioid peptides in spermatogenesis (39).

**Endocrinal causes**

**Figure 1:**
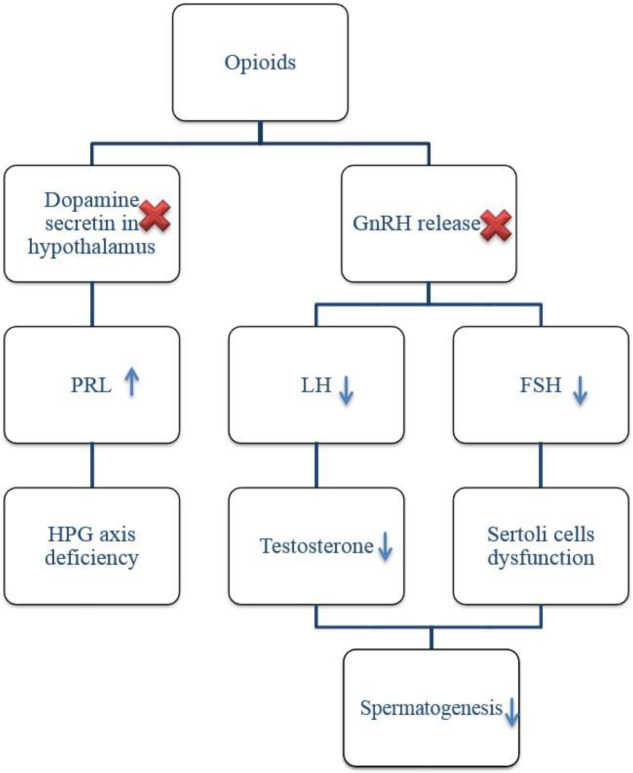
Opioids could inhibit the hypothalamus with either decreased GnRH secretion or level of dopamine which results in HPG impairment and abnormal spermatogenesis

Only 2% of patients with abnormal spermogram have been reported with an initial endocrine impairment as the main cause of infertility (40). A healthy HPG axis is essential to maintain balanced spermatogenesis. Gonadotropin-releasing hormones (GnRH) are released from the hypothalamus and induce a certain nucleus in the anterior lobe of the pituitary gland which results in gonadotropin secretion including follicle-stimulating hormone (FSH) and (LH) (41). LH induces Leydig cells to release testosterone while FSH affects the Sertoli cells which guard the developing sperm cells. Therefore, the function of gonads may be disturbed by a decrease of gonadotropin hormone secretion, lack of proper reaction toward gonadotropins, or abnormal non-gonadal endocrinal impacts on the HPG axis (3). It could be assumed that numerous studies regarding infertility found in addicted men are summarized in this part. However, presented reports in this field indicate obvious inconsistencies. The effect of illicit drugs on the HPG axis is summarized in Table 2 and Figure 1.

***Marijuana***

Several authors have studied the presence of cannabinoid receptors and their relation with hypothalamus neurons, plus marijuana components controlling GnRH release through the Gamma-aminobutyric acid (GABA) system and some other systems (42). According to these factors, the adverse effects of the drugs may be due to a disturbance in the HPG axis (43). A group of Japanese authors (2005) have determined marijuana impacts being similar to estrogens (44). This activity could restrain the secretion of GnRH through negative feedback. Similarly, a cannabinoid treatment in male rhesus monkeys can lead to a decreased basic level of prolactin (PRL) (45). In human studies, there are several controversial reports in this field. Although the expression of cannabinoid receptors in the anterior pituitary gland could account for the impaired HPG axis and a consequential reduction in LH and lower level of blood testosterone has been reported by researchers studying marijuana addicts, there can be found reports regarding the healthy HPG axis in consumers (46).

***Opioids***

Although morphine treatment of rats can change the medial eminence neurons (47), no structural change is observed in the human HPG axis after consuming opioids (48). The amount of PRL in the guinea pig (49) and human beings (50) treated with opioids will show a significant increase. Also, George has reviewed animal experiments in his book Narcotic Drugs: Biochemical Pharmacology, indicating that morphine will restrain the pituitary gland’s gonadotropin secretion, LH in particular. This pathway is intermediated by the hypothalamus and a reduction of GnRH production or release. Regarding human studies, researchers reported a reduction of basic gonadotropin levels in heroin administrations. GnRH treatment in this study indicated that compared with the control group, the addicted group showed a significantly lower response to the used hormone. The relative blocking of gonadotropin secretion in the pituitary level will induce the pituitary-testicular to detect and a long-term discharge of GnRH, which can be the reason for lower response to GnRH. Safarinejad *et al*. (2013) determined reductions in testosterone, free testosterone, and LH, plus a constant level of FSH measured in opioid-addicted men (38). A one-month spinal opioid injection resulted in decreased testosterone and free testosterone level in patients but no significant impacts were observed on PRL, LH, FSH, and sex-hormone-binding globulin (51). A pilot study on two detoxified male heroin addicts indicated that a 10 mg intravenous injection of heroin can cause a rapid drop of blood LH which is followed by a reduction of blood testosterone after four hours, while the same injection wouldn’t change the plasma level of FSH (48). A study on male heroin addicts (1979) who consumed over 150 mg heroin per day illustrated that the level of blood heroin has a negative relation with the concentration of testosterone and dihydrotestosterone, while the level of these two hormones will return to the initial stage when the plasma heroin is reduced. This study did not provide results indicating alterations in other gonadotropins including androstenedione (A), LH, and FSH (52). Another group of researchers reported constant levels of estradiol, LH, FSH, PRL, and testosterone in heroin addicts (53). Decreased testosterone and its relation with opioids are described in two different pathways: a) GnRH inhibition (30) or b) HPG axis impairment due to opioid-induced PRL release (51). Opioids such as heroin suppress the secretion of dopamine in the hypothalamus, thus reducing the inhibition impact of dopamine on the release of PRL from the anterior pituitary gland. Therefore, a number of studies have reported an increased level of PRL in narcotic consumers (54). Although the existing data is not consistent, the tolerance and duration period of study could be among the reasons causing the differences in results (51).

The diversity among results could be attributed to the age, lifestyle (i. e., financial status, education, nutrition, and exercise) (53), clinical conditions, sampling conditions (e. g., how much has it been since the last consumption) (48), addiction conditions including duration of addiction, simultaneous consumption of various drugs that could lead to difficulties during the recognition of medicine effects on each hormonal change (55), drug purity, daily consumption, and the variation among drug metabolisms.

According to these factors, it could be concluded that despite the prominent adjusting role of gonadocorticoids on sexual activities, the variable depends on uncontrollable factors and its alteration will not provide a convenient sexual health measurement. 

**Abnormalities found in other organs of the male reproductive system**

The male reproductive system consists of testicles, an associated duct system, accessory sex glands, and the penis (31).

**Abnormalities of accessory sex glands**

The accessory glands of the male reproductive system include seminal vesicles, prostate, and bulbourethral glands (31). Few studies have been conducted regarding the impact of illicit drugs on accessory sex glands.

***Marijuana***

THC reduces the semen-production-related enzymatic actions of the prostate in a dose-dependent manner (56). 

**Erection and ejaculation disorders**

According to the reports, ejaculation disturbances are found in 20–30% of men, which is common among infertile men (4). This disorder could be categorized into four classes consisting of premature ejaculation, retarded ejaculation, retrograde ejaculation, and failure of ejaculation (3). 

Although it’s most likely that hormonal suppression is the main reason behind the change in sexual behaviors of the addicted men, erection disorders could play the main role in reduced sexual behavior (57). Erection failure is another common sexual issue among men from which 10 to 20 percent of men suffer (58). The effect of illicit drugs on erection and ejaculation is presented in Table 3, briefly.

***Marijuana***

Adverse effects of cannabinoids on primates include reduced sexual arousal and erection disorders which remain even after withdrawal. In human studies some authors have demonstrated that cannabis was related to premature ejaculation (59), reduced libido (60) with other erection disorders (59), along with a relationship between cannabis and vascular disturbances of erection affecting the endothelial function, while another study (2010) found no significant relationship between cannabis consumption and sexual activity (61).

***Opioids***

Increased intromission was observed in morphine-treated rats as an inhibitor effect of opioids on sexual reflexes (59). In human beings like tramadol, opioids could affect male fertility when used as a painkiller or to cure premature ejaculation. Some authors have reported reduced libido, sexual inadequacy, and delayed ejaculation in heroin or methadone-addicted men who are withdrawing (62). Similarly, month-long spinal injection of morphine and hydromorphone can result in disorders of erectile function and reduced libido (51).

**Microbiology of semen and infections of the male reproductive system**

Infections in the male reproductive system may cause damage to male fertility directly or to the testis, the vas deferens, and accessory sex glands (2). Clinical symptoms of infections in the male reproductive system include pain, fever, and blood presence in the semen. The indication of clinically asymptomatic infection is an increased number of leukocytes in the semen where it is known as pyospermia or leukocytospermia (63). Accumulating leukocytes in the semen is generally an indicator of infection; however, it should be taken into account that neutrophils are to be found in the semen of elderly men diagnosed with benign prostate enlargement. Leukocytospermia should be under medical treatment all the same (3). 

***Opioids***

Few reports have been presented microbiological assessments on addicted men (64) plus leukocytospermia in heroin administration (53) are mainly studied in this field. According to the recent hypothesis, leukocytospermia may result in infertility through microenvironmental changes in semen. This issue will be explained in the “molecular alterations of semen and sperm” part.

**Semen analysis**

A semen analysis or seminogram studies the most important characteristics of semen and sperm which include sperm motility, concentration, and morphology standardized by WHO. The analytical results are affected by sampling methods and measurement accuracies (65). The impact of illicit drugs on seminogram has been summarized in Table 4.

***Marijuana***

Abnormal sperm morphology has been reported in rabbits and mice (66). Another adverse effect reported in mice is the decrease in sperm concentration. In human studies decreased semen volume, sperm concentration, morphology, motility, and fertility characteristics of sperm (30) along with sperm hyperactivity have been reported in cannabis-addicted men. An *in vitro* exposure to THC indicated decreased progressive motility and spontaneous acrosome reaction in human sperm (67). The expression of type I of the cannabinoid receptors and fatty acid amide hydrolase in elongating spermatids and spermatozoa indicated that endogenous cannabinoids are included in spermatogenesis and sperm physiology (68) which somewhat can account for the resulting changes.

***Opioids***

Heroin can cause a significant decrease in sperm motility and viability (15). Iranian Kerack has destructive effects on the mouse sperm characteristics including sperm concentration, viability, progressive motility, and morphology. Most observed abnormal morphology consists of twisted tail and midpiece, pinhead, and other head abnormalities (69). In humans, heroin consumption may result in most abnormal morphology, particularly in the head and tail of the sperm. There is a direct relation between sperm morphology and duration of addiction (53). Motility is another prominent sperm parameter that is reduced under heroin impacts (53, 70). These two parameters could be alternated under the impact of other opioids, as well (38).

***Cocaine***

decreased sperm count to under 20 million sperm cells per ml will be observed through the initial two years of cocaine abuse followed by decreased sperm motility and increased abnormal morphology in male addicts who have been consuming cocaine for over four years. These observations indicate that cocaine has adverse effects on fertility (22).

**Figure 2:**
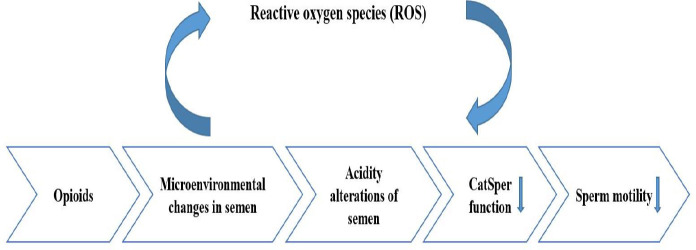
Microenvironmental changes in the genital tract of opioid addicts can lead to seminal pH alteration, and genetic abnormality in spermatozoa affect sperm cell motility

**Figure 3:**
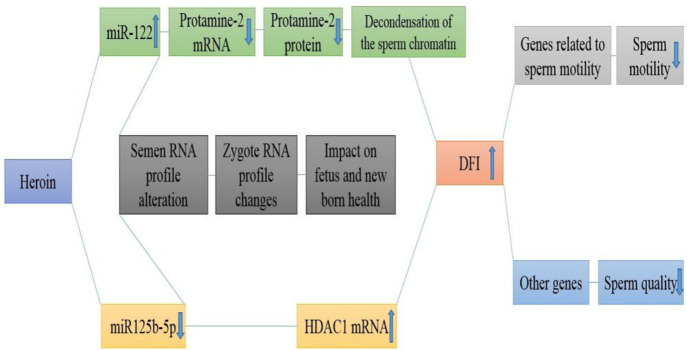
Heroin can impair the sperm's genetic and epigenetic factors related to nuclear condensation and motility, increased DFI percentage, and alter the RNA profile transmitted to the fetus

Molecular alterations of semen and sperm-related paper are gathered in Table 5, Figure 2, and Figure 3.

**Free radicals in the semen**

A misbalance among free oxygen and antioxidants present in the semen may result in oxidative stress which is the most known male infertility factor in males among non-genetic reasons (71). Oxidative stress is induced by increased production of reactive oxygen species (ROS) (72). ROS is necessary for capacitation, acrosome reaction, and fertilization. However, decreased removal and production of ROS will result in DNA damage and harm the integrity of the sperm plasma membrane thus reducing fertility (73).

***Opioids***

opioid consumption can result in decreased antioxidant capability of semen and increased ROS (38). Studies of the differentiated SH-SY5Y cells indicate morphine being a ROS inducer and the amount of ROS depends on the amount of morphine and incubation time. The μ-opioid receptor is the first site of action for the most commonly used opioids and messenger RNA (mRNA) level of the µ receptor in these cells is decreased according to morphine concentration. The decreased transcript of this receptor is related to the increased exogenous antioxidants and ROS of the cells (74). Thus, in addition to bonding with the µ receptor, morphine also interferes with ROS production in cellular function. 

**Sperm plasma membrane**

Studies of endogenous opioid receptors, enkephalin degrading enzymes, and membrane channels have been reviewed in this part. 

**Endogenous opioid receptors**

Endogenous receptors perform based on three main receptors ᵟ, ᵏ, and µ. Endorphine and enkephalin are endogenous peptides that are found in the male reproductive system (75). The presence of receptors for these peptides along the entire plasma membrane of human sperm (head, midpiece, and tail) was first discovered in 2006 by Agirregoitia *et al*. Also, the expression of ᵏ receptor in the cytoplasm was found. Although ᵟ receptors were observed on the plasma membrane, the mRNA expression level of this receptor was unmeasurable in mature sperm cells (76). This can indicate expression of this receptor during spermatogenesis. Similarly, the presence of ᵏ and µ transcripts in the RNA profile of mature spermatozoids can interfere with the development of primary zygotes.

***Opioids***

Incubation of human sperm using morphine (an agonist of µ receptors) will increase the number of immotile sperm cells, while naloxone (antagonist of µ receptors) induces an increase in motile sperm cells (76). Heroin is another agonist of µ receptors. Asthenospermia is considered the most significant adverse effect of heroin on addicted men (53, 70), thus one of the pathways of drug effects may be mediated by endogenous receptors (70). Enkephalin is more likely to bond with ᵟ receptors than to µ receptors. High doses of enkephalin will reduce motility, while a low dose is necessary to retain sperm motility (77). It has been claimed that enkephalin not bonding to the ᵟ receptor is due to the receptor being occupied by antagonists, where the inhibitor effect of ᵟ receptor antagonists such as naltrindole on sperm motility is described. The inhibitor effect of high enkephalin doses will justify the attachment of this peptide to the µ receptors. A low dosage of morphine (0.1 µM) will reduce sperm motility while the higher doses leave no effect (1–10 µM). In these conditions, morphine will form bonds with µ and ᵟ receptors, further inducing the ᵟ receptor which is necessary to retain sperm motility (76).

**Enkephalin degrading enzymes**

Two significant enzyme pathways that hydrolyze enkephalin consist of glycine-phenylalanine hydrolase performed by enkephalinase and cleavage of the tyrosine-glycine bond by N aminopeptidase. The activity of these two enzymes is much higher in the semen compared with the rest of the tissues. The presence of aminopeptidases N in the head, neck, and along the tail of the sperm is identified as well as in different fractions of semen. However, enkephalinase is found in a very restricted area of a few sperm cells and fractions of semen. The inhibition of the activity of the enzymes will reduce sperm motility (78).

***Opioids***

A study reported reduced transcript and protein of aminopeptidase N and reduced mRNA expression level of enkephalinase in mature sperm cells of heroin-addicted men. The reduction of aminopeptidase N was found to be relevant to the duration of addiction (70). The limited expression of the enzyme in certain regions of capacitated sperm cells indicates the steady level of aminopeptidase N in addicted and healthy men (70).

**Membrane channels**

Ionic channels, calcium channels, in particular, have a significant role in various stages of mammalian fertilization including sperm capacitation, acrosome reaction, and sperm motility (79). An example of calcium transferring channels could be CatSper 1 to 4 (79) which are exclusively expressed in sperm cells and have a significant role in sperm function and motility. CatSper 1 to 4 form a tetramer that is located in the plasma membrane of the principal piece of the sperm tail (80).

***Opioids***

Decreased expression of CatSper 1-4 mRNAs and relation with the dose-dependent motility of mice treated with Iranian Kerack was demonstrated (69). According to the acidity alterations of semen in relation to drug abuse (53) and the importance of pH in the performance of this channel, it could be concluded that semen pH alterations impact the channel function. An increased opioid-induced ROS may cause opioids to disturb protein expression, CatSpers included (69).

**Sperm nucleus**

Accurate chromatin packaging can affect morphology and motility of sperm cells. Compared with the somatic cell nucleus, the sperm nucleus is tenfold compressed, mediated by histone protein replacement by protamines. Factors affecting the procedure include histone deacetylase enzymes (HDCA) which are included in the decondensation of chromatin (81), along with correct genes and proteins of protamines which eventually will replace the histones (82). This transferring process is an indicator of sperm nucleus maturation. About 80 to 85% of histone present in the mature human sperm is replaced with protamine (83).

***Opioids***

According to the studies, consumption of different opioids, heroin, in particular, is related to increased nucleus maturation disorders (53) and DNA fragmentation index (DFI) of sperm cells. Nazmara *et al*. have studied the male infertility factors at the molecular level as pioneers, considering the health of sperm heads (the sperm nucleus) and protamine gene expression and proteins, particularly. Reduced gene expression and protamine-2 protein in sperm cells indicate heroin affecting protamine production. Similarly, increased expression level of HDAC1, decreased HDAC11 transcripts, and increased DFI% in male heroin addicts, indicate that alterations in the HDAC1 expression level will induce abnormal decondensation of the sperm chromatin in heroin consumers, thus increasing the risk of DNA damage (DFI). The increasing DFI% being directly related to the number of HDAC1 transcripts has confirmed the suggested hypothesis. There are few reports regarding the deacetylase functions of HDAC11. The ability of this enzyme to deacetylase histone directly has not been yet confirmed (84). According to the decreased transcription of HDAC11 and the negative relation with DFI% and leukocytospermia, it has been suggested that HDAC11 might not interfere with histone deacetylation but functioning through its impact on the immune system (84).

**The ribonucleic acid content of semen**

MicroRNAs (miRNA) are considered one kind of the most important non-coding small RNAs present in the RNA profile of semen which regulate the gene expression in spermatogenesis, taking part in successful fertility with transport to the oocyte during fertilization (85). Recently, these molecules have been used as novel noninvasive biomarkers to identify various types of infertility. The relation between the expression level of miRNAs with fertility and fetus health has been considered by several authors (86, 87).

***Opioids***

 The study conducted on male heroin addicts indicated an increased miR-122 expression level and its relation to the decreased protamine-2 transcripts, along with a decreased miR-125b-5p expression level and its relation with increased HDAC1 transcripts. The results of these studies revealed that heroin can affect the Ribonucleic acid content of semen; also miRNAs have a regulating role in gene expression as an epigenetic factor.

**Table 1:** Effects of illicit drugs on different species in primary testicular disease

Parameters	Illicit drugs	Species	References
Deficiency in Leydig cell function	Marijuana	Rat	Wenger *et al*. (88)
	Opioids	Human	Bolelli *et al*. (52)
Induced apoptosis in seminiferous tubules	Amphetamines	Mouse	Yamamoto *et al*. (89)
	Cocaine	Human	Sansone *et al*. (30)
Decreased diameter of seminiferous tubules	Marijuana	Dog	Dixit *et al*. (24)
Decreased cell adhesions	Marijuana	Dog	Dixit *et al*. (24)
Reduced epithelial thickness	Iranian Kerack	Mouse	Amini *et al*. (69)
	Methadone	Rat	Heidari *et al*. (27)
	Tramadol	Rat	El-Ghawet *et al*. (28)
	Morphine	Rat	Takzare *et al*. (29)
Degenerated tubules	Ecstasy	Rat	Sansone *et al*. (30)
Deficient spermatogenesis and sperm functionality	Marijuana	Vertebrates	Bari *et al*. (19)
	Marijuana	Mouse	Wenger *et al*. (88)
	Marijuana	Dog	Dixit *et al*. (24)
	Marijuana	Human	Alvarez *et al*. (20), Dai *et al*. (36)
	Heroin	Mouse	Simin F, Zahra T. (15)
	Morphine	Rat	Takzare *et al*. (29)
	Opioids	Human	Safarinejad *et al*. (38)

HPG: hypothalamic-pituitary-gonadal; GnRH: gonadotropin-releasing hormones

**Table 2:** Effects of different kinds of illicit drugs on the parameters related to the HPG axis

Parameters		Illicit drugs	Species	References
GnRH release	Inhibition	Marijuana	Mouse	Farkas *et al*. (42)
		Marijuana	Rat	Watanabe *et al*. (44)
Prolactin	Constant	Opioids	Human	Paice *et al*. (51), Nazmara *et al*. (53)
LH	Decreased	Marijuana	Human	Diamond *et al*. (55)
		Opioids	Human	Safarinejad *et al*. (38), Mirin *et al*. (48)
	Constant	Opioids	Human	Paice *et al*. (51), Bolelli *et al*. (52), Nazmara *et al*. (53)
FSH	Constant	Opioids	Human	Safarinejad *et al*. (38), Paice *et al*. (51), Mirin *et al*. (48), Bolelli *et al*. (52), Nazmara *et al*. (53)
Estradiol	Constant	Heroin	Human	Nazmara *et al*. (53)
Androstenedione	Constant	Heroin	Human	Bolelli *et al*. (52)
Testosterone	Decreased	Marijuana	Human	Diamond *et al*. (55)
		Opioids	Human	Safarinejad *et al*. (38), Paice *et al*. (51), Mirin *et al*. (48), Bolelli *et al*. (52)
	Constant	Heroin	Human	Nazmara *et al*. (53)

HPG: hypothalamic-pituitary-gonadal; GnRH: gonadotropin-releasing hormones; Lh: luteinizing hormone; FSH: follicle-stimulating hormone

**Table 3:** Effects of illicit drugs on erection and ejaculation in the human

Parameters		Illicit drugs	Species	References
Erection disorders		Marijuana	Human	Mialon *et al*. (59), Park *et al*. (60)
Erection disorders		Morphine	Human	Paice *et al*. (51)
Ejaculation		Marijuana	Human	Mialon *et al*. (59)
Reduced libido		Marijuana	Human	Park *et al*. (60)
		Heroin	Human	Paice *et al*. (51)
Sexual activity		Marijuana	Human	Smith *et al*. (61)

**Table 4:** Impacts of illicit drugs on the parameters related to sperm

Parameters		Illicit drugs	Species	References
Abnormal sperm morphology		Marijuana	Human	Sansone *et al*. (30)
		Iranian Kerack	Mouse	Amini *et al*. (69)
		Opioids	Human	Safarinejad *et al*. (38), Nazmara *et al*. (53)
Decrease in sperm concentration		Iranian Kerack	Mouse	Amini *et al*. (69)
Decreased sperm motility	Total	Marijuana	Human	Sansone *et al*. (30)
		Heroin	Human	Nazmara *et al*. (53), Rezaei-Mojaz *et al*. (70)
	Progressive	Marijuana	Human	Whan *et al*. (67)
		Opioids	Mouse	Simin *et al*. (15), Amini *et al*. (69)
		Opioids	Human	Safarinejad *et al*. (38)
Sperm viability		Opioids	Mouse	Simin *et al*. (15), Amini *et al*.(69)
Decreased semen volume		Marijuana	Human	Sansone *et al*. (30)

**Table 5:** Molecular alterations of semen and sperm as a consequence of illicit drug consumption

	Parameters		Illicit drugs			Species	References
Semen	Free radicals	Decreased antioxidant capability	Opioids			Human	Safarinejad *et al*. (38)
		Increased ROS	Morphine			Human (cell line)	Safarinejad *et al*. (38), Ma *et al*. (74)
Sperm plasma membrane	ᵟ ,ᵏ, and µ receptors	Decreased sperm motility		Agonist of µ receptors	Morphine	Human (*in vitro*)	Agirregoitia *et al*. (76)
					Heroin	Human	Nazmara *et al*. (53), Rezaei-Mojaz *et al*. (70)
				Antagonist of ᵟ receptors	Naltrindole	Human	Agirregoitia *et al*. (76)
		Increased sperm motility	Antagonist of µ receptors		Naloxone	Human	Agirregoitia *et al*. (76)
	Enkephalin degrading enzymes	Decreased aminopeptidase N (mRNA, Protein)	Heroin			Human	Rezaei-Mojaz *et al*. (70)
		Decreased enkephalinase (mRNA)	Heroin			Human	Rezaei-Mojaz *et al*. (70)
	Decreased expression of CatSper 1 - 4 mRNAs		Iranian Kerack			Mice	Amini *et al*. (69)
Sperm nucleus	Histone-to-protamine Transition deficiency		Heroin			Human	Nazmara *et al*. (53)
	Increased DFI %		Opioids			Human	Safarinejad *et al*. (38)
	Decreased protamine-2 (mRNA and Protein)		Heroin			Human	Nazmara *et al*. (11)
	Increased HDAC1		Heroin			Human	Nazmara *et al*. (85)
	Decreased HDAC11		Heroin			Human	Nazmara *et al*. (85)
	Increased miR-122		Heroin			Human	Nazmara *et al*. (11)
	Decreased miR-125b-5p		Heroin			Human	Nazmara *et al*. (11)

**Figure 4:**
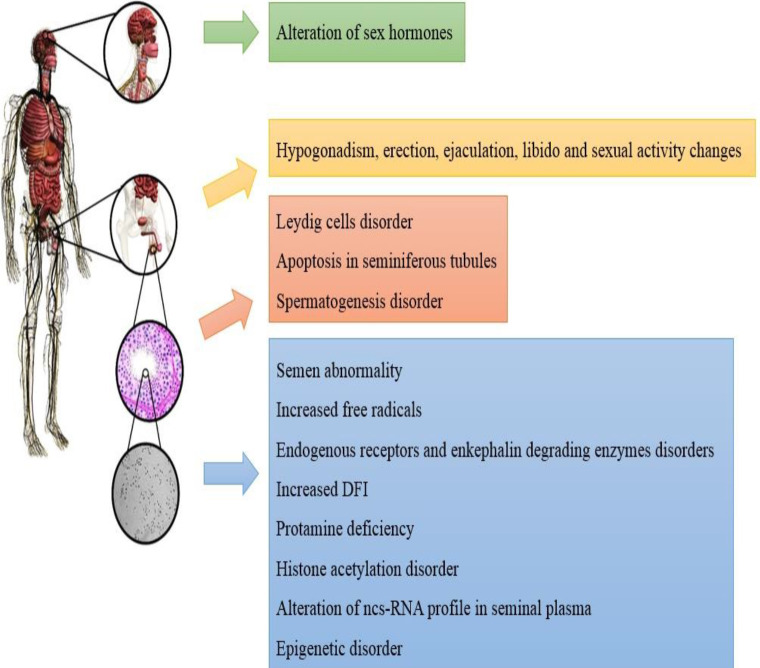
Structural and functional impairment, endocrinal disorders, changes in sexual behavior and semen analysis, and molecular alterations of semen in humans

**Figure 5:**
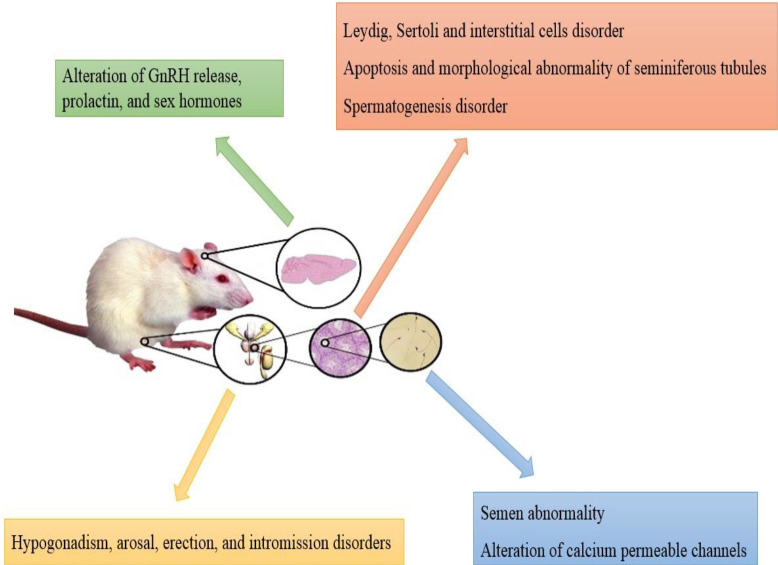
Structural and functional impairment, endocrinal disorders, changes in sexual behavior and semen analysis, and molecular alterations of semen in an animal model

## Conclusion

Narcotic drug use in childhood, pre-adolescent, juvenile, and adulthood of men may lead to male infertility. While clinical studies are limited by legal and ethical restrictions and multiple drug consumption, structural and functional impairment of testis, endocrinal disorders, changes in sexual behavior and semen analysis, as well as molecular alterations of semen and spermatozoa have been reported in human and animal studies (Figures 4 and 5). This narcotic drug addiction can be considered one of the risk factors in male infertility. Using these data for the cessation of drug consumption during infertility treatment can increase fertility rates in reproductive medicine centers.

## References

[1] Agarwal A, Mulgund A, Hamada A, Chyatte MR (2015). A unique view on male infertility around the globe. Reprod Biol Endocrinol..

[2] Fijak M, Pilatz A, Hedger MP, Nicolas N, Bhushan S, Michel V (2018). Infectious, inflammatory and ‘autoimmune’male factor infertility: How do rodent models inform clinical practice?. Hum Reprod Update..

[3] Jequier AM (2008). Male infertility: A guide for the clinician.

[4] Porst H, Montorsi F, Rosen RC, Gaynor L, Grupe S, Alexander J (2007). The premature ejaculation prevalence and attitudes (PEPA) survey: Prevalence, comorbidities, and professional help-seeking. Eur Urol.

[5] Bjorndahl L, Barratt CL, Mortimer D, Jouannet P (2016). ‘How to count sperm properly’: checklist for acceptability of studies based on human semen analysis. Hum Reprod.

[6] Choobineh H, Kazemi M, Sadighi Gilani MA, Heydari T, Shokri S, Bazrafkan M (2018). Testosterone reduces spinal cord injury-induced effects on male reproduction by preventing CADM1 Defect. Cell J.

[7] Wang A, Stormont I, Siddiqui MM (2017). A review of imaging modalities used in the diagnosis and management of scrotal trauma. Curr Urol Rep..

[8] Brauner EV, Nordkap L, Priskorn L, Hansen AM, Bang AK, Holmboe SA (2020). Psychological stress, stressful life events, male factor infertility, and testicular function: a cross-sectional study. Fertil Steril..

[9] Marcho C, Oluwayiose OA, Pilsner JR (2020). The preconception environment and sperm epigenetics. Andrology.

[10] Ladjouze A, Donaldson M (2019). Primary gonadal failure. Best Pract Res Clin Endocrinol Metab.

[11] Nazmara Z, Najafi M, Movahedin M, Zandiyeh Z, Shirinbayan P, reza Asgari H (2020). Correlation between protamine-2 and miRNA-122 in sperm from heroin-addicted men: A case-control study. Urol J.

[12] Cicero TJ, O’Connor L, Nock B, Adams ML, Miller BT, Bell RD (1989). Age-related differences in the sensitivity to opiate-induced perturbations in reproductive endocrinology in the developing and adult male rat. J Pharmacol Exp Ther.

[13] Richards JR, Wang CG, Fontenette RW, Stuart RP, McMahon KF, Turnipseed SD (2020). Rhabdomyolysis, methamphetamine, amphetamine and MDMA Use: Associated factors and risks. J Dual Diagn.

[14] Yilmaz B, Konar V, Kutlu S, Sandal S, Canpolat S, Gezen MR (1999). Influence of chronic morphine exposure on serum LH, FSH, testosterone levels, and body and testicular weights in the developing male rat. Arch Androl.

[15] Simin F, Zahra T (2007). Effect of heroin used in Iran on male fertility of mice. Int J Pharmacol.

[16] Xiong X, Zhang L, Fan M, Han L, Wu Q, Liu S (2019). β-Endorphin induction by psychological stress promotes leydig cell apoptosis through p38 MAPK pathway in male rats. Cells.

[17] Bawor M, Bami H, Dennis BB, Plater C, Worster A, Varenbut M (2015). Testosterone suppression in opioid users: a systematic review and meta-analysis. Drug Alcohol Depend.

[18] Semet M, Paci M, Saïas-Magnan J, Metzler-Guillemain C, Boissier R, Lejeune H (2017). The impact of drugs on male fertility: A review. Andrology.

[19] Bari M, Battista N, Pirazzi V, Maccarrone M (2011). The manifold actions of endocannabinoids on female and male reproductive events. Front Biosci.

[20] Alvarez S (2015). Do some addictions interfere with fertility?. Fertil Steril..

[21] Nudmamud-Thanoi S, Sueudom W, Tangsrisakda N, Thanoi S (2016). Changes of sperm quality and hormone receptors in the rat testis after exposure to methamphetamine. Drug Chem Toxicol.

[22] Samplaski MK, Bachir BG, Lo KC, Grober ED, Lau S, Jarvi KA (2014). Cocaine use in the infertile male population: a marker for conditions resulting in subfertility. Curr Urol.

[23] González B, Pantoja CRG, Sosa MH, Vitullo AD, Bisagno V, González CR (2018). Cocaine alters the mouse testicular epigenome with direct impact on histone acetylation and DNA methylation marks. Reprod Biomed Online.

[24] Dixit VP, Gupta CL, Agrawal M (1977). Testicular degeneration and necrosis induced by chronic administration of cannabis extract in dogs. Endokrinologie.

[25] Tajaddini Mahani S, Behnam B, Abbassi M, Asgari H, Nazmara Z, Shirinbayan P (2016). Tsga10 expression correlates with sperm profiles in the adult formalin-exposed mice. Andrologia.

[26] Akhgari M, Jokar F, Bahmanabadi L, Aleagha AE (2012). Street-level heroin seizures in Iran: A survey of components. J Subst Use.

[27] Heidari Z, Mahmoudzadeh-Sagheb H, Kohan F (2012). A quantitative and qualitative study of rat testis following administration of methadone and buprenorphine. Int J High Risk Behav Addict.

[28] El-Ghawet HA (2015). Effects of tramadol on the reproductive function of wistar albino rats. Eur J Exp Biol.

[29] Takzare N, Samizadeh E, Shoar S, Majidi Zolbin M, Naderan M, Lashkari A (2016). Impacts of morphine addiction on spermatogenesis in rats. Int J Reprod Biomed.

[30] Sansone A, Di Dato C, de Angelis C, Menafra D, Pozza C, Pivonello R (2018). Smoke, alcohol and drug addiction and male fertility. Reprod Biol Endocrinol.

[31] Ross MH, Pawlina W (2019). Histology: A Text and Atlas: With correlated cell and molecular biology.

[32] Mai HN, Chung YH, Shin E-J, Jeong JH, Jung TW, Sharma N (2019). Overexpression of glutathione peroxidase-1 attenuates cocaine-induced reproductive dysfunction in male mice by inhibiting nuclear factor κB. Chem Biol Interact.

[33] Du Plessis SS, Agarwal A, Syriac A (2015). Marijuana, phytocannabinoids, the endocannabinoid system, and male fertility. J Assist Reprod Genet.

[34] Rajanahally S, Raheem O, Rogers M, Brisbane W, Ostrowski K, Lendvay T (2019). The relationship between cannabis and male infertility, sexual health, and neoplasm: a systematic review. Andrology.

[35] Barchi M, Innocenzi E, Giannattasio T, Dolci S, Rossi P, Grimaldi P (2019). Cannabinoid receptors signaling in the development, epigenetics, and tumours of male germ cells. Int J Mol Sci.

[36] Dai JB, Wang ZX, Qiao ZD (2015). The hazardous effects of tobacco smoking on male fertility. Asian J Androl..

[37] Vuong C, Van Uum SH, O’Dell LE, Lutfy K, Friedman TC (2010). The effects of opioids and opioid analogs on animal and human endocrine systems. Endocr Rev.

[38] Safarinejad MR, Asgari SA, Farshi A, Ghaedi G, Kolahi AA, Iravani S (2013). The effects of opiate consumption on serum reproductive hormone levels, sperm parameters, seminal plasma antioxidant capacity and sperm DNA integrity. Reprod Toxicol.

[39] Nargund VH (2015). Effects of psychological stress on male fertility. Nat Rev Urol.

[40] Sengupta P, Arafa M, Elbardisi H Hormonal regulation of spermatogenesis. Molecular Signaling in Spermatogenesis and Male Infertility: CRC Press.

[41] Oduwole OO, Peltoketo H, Huhtaniemi IT (2018). Role of follicle-stimulating hormone in spermatogenesis. Front Endocrinol.

[42] Farkas I, Kallo I, Deli L, Vida B, Hrabovszky E, Fekete C (2010). Retrograde endocannabinoid signaling reduces GABAergic synaptic transmission to gonadotropin-releasing hormone neurons. Endocrinology.

[43] Huizink AC, Ferdinand RF, Ormel J, Verhulst FC (2006). Hypothalamic-pituitary-adrenal axis activity and early onset of cannabis use. Addiction.

[44] Watanabe K, Motoya E, Matsuzawa N, Funahashi T, Kimura T, Matsunaga T (2005). Marijuana extracts possess the effects like the endocrine disrupting chemicals. Toxicology.

[45] John WS, Martin TJ, Nader MA (2020). Cannabinoid modulation of food-cocaine choice in male rhesus monkeys. J Pharmacol Exp Ther.

[46] Borowska M, Czarnywojtek A, Sawicka-Gutaj N, Woliński K, Płazińska MT, Mikołajczak P (2018). The effects of cannabinoids on the endocrine system. Endokrynol Pol..

[47] Wehbeh L, Dobs AS (2020). Opioids and the Hypothalamic-Pituitary-Gonadal (HPG) Axis. J Clin Endocrinol Metab..

[48] Mirin SM, Mendelson JH, Ellingboe J, Meyer RE (1976). Acute effects of heroin and naltrexone on testosterone and gonadotropin secretion: A pilot study. Psychoneuroendocrinology.

[49] Weems PW, Witty CF, Amstalden M, Coolen LM, Goodman RL, Lehman MN (2016). κ-Opioid receptor is colocalized in GnRH and KNDy cells in the female ovine and rat brain. Endocrinology..

[50] Farag A, Basha M, Amin S, Elnaidany N, Elhelbawy N, Mostafa M (2018). Tramadol (opioid) abuse is associated with a dose-and time-dependent poor sperm quality and hyperprolactinaemia in young men. Andrologia.

[51] Paice JA, Penn RD, Ryan WG (1994). Altered sexual function and decreased testosterone in patients receiving intraspinal opioids. J Pain Symptom Manage.

[52] Bolelli G, Lafisca S, Flamigni C, Lodi S, Franceschetti F, Filicori M (1979). Heroin addiction: Relationship between the plasma levels of testosterone, dihydrotestosterone, androstenedione, LH, FSH, and the plasma concentration of heroin. Toxicology.

[53] Nazmara Z, Najafi M, Rezaei-Mojaz S, Movahedin M, Zandiyeh Z, Shirinbayan P (2019). The effect of heroin addiction on human sperm parameters, histone-to-protamine transition, and serum sexual hormone levels. Urol J.

[54] Spasovska Trajanovska A, Vujovic V, Ignjatova L, Janikevik Ivanovska D, Chibishev A (2013). Sexual dysfunction as a side effect of hyperprolactinemia in methadone maintenance therapy. Med Arch.

[55] Diamond F Jr, Ringenberg L, MacDonald D, Barnes J, Hu CS, Duckett G (1986). Effects of drug and alcohol abuse upon pituitary-testicular function in adolescent males. J Adolesc Health Care.

[56] Chakravarty I, Ghosh JJ (1981). Influence of cannabis and delta-9-tetrahydrocannabinol on the biochemistry of the male reproductive organs. Biochem Pharmacol..

[57] Rokach A (2019). The Effect of Psychological Conditions on Sexuality: A Review. Psychopharmacology.

[58] McCabe MP, Sharlip ID, Lewis R, Atalla E, Balon R, Fisher AD (2016). Incidence and prevalence of sexual dysfunction in women and men: a consensus statement from the fourth international consultation on sexual medicine 2015. J Sex Med.

[59] Mialon A, Berchtold A, Michaud PA, Gmel G, Suris JC (2012). Sexual dysfunctions among young men: prevalence and associated factors. J Adolesc Health.

[60] Park B, McPartland JM, Glass M (2004). Cannabis, cannabinoids and reproduction. Prostaglandins Leukot Essent Fatty Acids.

[61] Smith AM, Ferris JA, Simpson JM, Shelley J, Pitts MK, Richters J (2010). Cannabis use and sexual health. J Sex Med.

[62] Grover S, Mattoo SK, Pendharkar S, Kandappan V (2014). Sexual dysfunction in patients with alcohol and opioid dependence. Indian J Psychol Med.

[63] Vázquez-Martínez ER, García-Gómez E, Camacho-Arroyo I, González-Pedrajo B (2018). Sexual dimorphism in bacterial infections. Biol Sex Differ.

[64] Evelina L, Eugen C (2018). Tuberculosis evolution and treatment outcome in drug addicted patients. Moldovan Med J.

[65] Lozano GM, Bejarano I, Espino J, González D, Ortiz Á, García JF (2009). Density gradient capacitation is the most suitable method to improve fertilization and to reduce DNA fragmentation positive spermatozoa of infertile men. Anatolian J Obstet Gynecol.

[66] Murphy SK, Itchon-Ramos N, Visco Z, Huang Z, Grenier C, Schrott R (2018). Cannabinoid exposure and altered DNA methylation in rat and human sperm. Epigenetics.

[67] Whan LB, West MC, McClure N, Lewis SE (2006). Effects of delta-9-tetrahydrocannabinol, the primary psychoactive cannabinoid in marijuana, on human sperm function in vitro. Fertil Steril.

[68] Cacciola G, Chioccarelli T, Ricci G, Meccariello R, Fasano S, Pierantoni R (2008). The endocannabinoid system in vertebrate male reproduction: a comparative overview. Mol Cell Endocrinol.

[69] Amini M, Shirinbayan P, Behnam B, Roghani M, Farhoudian A, Joghataei MT (2014). Correlation between expression of CatSper family and sperm profiles in the adult mouse testis following Iranian Kerack abuse. Andrology.

[70] Rezaei-Mojaz S, Nazmara Z, Najafi M, Movahedin M, Zandieh Z, Shirinbayan P (2019). Evaluation of enkephalin-degrading enzymes in sperm from heroin-addicted men. Int J Fertil Steril.

[71] Ebrahimi B, Forouzanfar F, Azizi H, Khoshdel-Sarkarizi H, Sadeghnia H, Rajabzadeh A (2020). Effect of electroacupuncture and glibenclamide on blood glucose level and oxidative stress parameters in streptozotocin-induced diabetic rats and possible human implications. Acupunct Electro-Therapeutics Res..

[72] Bafadam S, Beheshti F, Khodabakhshi T, Asghari A, Ebrahimi B, Sadeghnia HR (2019). Trigonella foenum-graceum seed (Fenugreek) hydroalcoholic extract improved the oxidative stress status in a rat model of diabetes-induced memory impairment. Horm Mol Biolo Clin Investig.

[73] Lombardo F, Sansone A, Romanelli F, Paoli D, Gandini L, Lenzi A (2011). The role of antioxidant therapy in the treatment of male infertility: An overview. Asian J Androl.

[74] Ma J, Yuan X, Qu H, Zhang J, Wang D, Sun X (2015). The role of reactive oxygen species in morphine addiction of SH-SY5Y cells. Life Sci.

[75] Sayed RK, Mokhtar DM, Fernández-Ortiz M, Escames G, Acuña-Castroviejo D (2019). Retinoid-related orphan nuclear receptor alpha (RORα)-deficient mice display morphological testicular defects. Lab Invest.

[76] Agirregoitia E, Valdivia A, Carracedo A, Casis L, Gil J, Subiran N (2006). Expression and localization of delta-, kappa-, and mu-opioid receptors in human spermatozoa and implications for sperm motility. J Clin Endocrinol Metab.

[77] Wang Y, Zhao X, Gao X, Gan Y, Liu Y, Zhao X (2017). Original endomorphin-1 analogues exhibit good analgesic effects with minimal implications for human sperm motility. Bioorg Med Chem Lett.

[78] Subiran N, Agirregoitia E, Valdivia A, Ochoa C, Casis L, Irazusta J (2008). Expression of enkephalin-degrading enzymes in human semen and implications for sperm motility. Fertil Steril.

[79] Singh AP, Rajender S (2015). CatSper channel, sperm function and male fertility. Reprod Biomed Online.

[80] Qi H, Moran MM, Navarro B, Chong JA, Krapivinsky G, Krapivinsky L (2007). All four CatSper ion channel proteins are required for male fertility and sperm cell hyperactivated motility. Proc Natl Acad Sci USA.

[81] Kim JH, Jee BC, Lee JM, Suh CS, Kim SH (2014). Histone acetylation level and histone acetyltransferase/deacetylase activity in ejaculated sperm from normozoospermic men. Yonsei Med J.

[82] Aoki VW, Liu L, Carrell DT (2005). Identification and evaluation of a novel sperm protamine abnormality in a population of infertile males. Hum Reprod.

[83] Miller D, Brinkworth M, Iles D (2010). Paternal DNA packaging in spermatozoa: more than the sum of its parts? DNA, histones, protamines and epigenetics. Reproduction.

[84] Cao J, Sun L, Aramsangtienchai P, Spiegelman NA, Zhang X, Huang W (2019). HDAC11 regulates type I interferon signaling through defatty-acylation of SHMT2. Proc Natl Acad Sci USA.

[85] Nazmara Z SP, Asgari HR, Ahadi R, Asgari F, Maki CB (2020). The epigenetic alterations of human sperm cells caused by heroin use disorder. Andrologia.

[86] Wang C, Yang C, Chen X, Yao B, Yang C, Zhu C (2011). Altered profile of seminal plasma microRNAs in the molecular diagnosis of male infertility. Clin Chem.

[87] Abu-Halima M, Backes C, Leidinger P, Keller A, Lubbad AM, Hammadeh M (2014). MicroRNA expression profiles in human testicular tissues of infertile men with different histopathologic patterns. Fertil Steril.

[88] Wenger T, Ledent C, Csernus V, Gerendai I (2001). The central cannabinoid receptor inactivation suppresses endocrine reproductive functions. Biochem Biophys Res Commun.

[89] Yamamoto Y, Yamamoto K, Hayase T, Abiru H, Shiota K, Mori C (2002). Methamphetamine induces apoptosis in seminiferous tubules in male mice testis. Toxicol Appl Pharmacol.

